# Comprehensive profiling of stem-like features in pediatric glioma cell cultures and their relation to the subventricular zone

**DOI:** 10.1186/s40478-023-01586-x

**Published:** 2023-06-16

**Authors:** Marc-Antoine Da-Veiga, Natacha Coppieters, Arnaud Lombard, Bernard Rogister, Virginie Neirinckx, Caroline Piette

**Affiliations:** 1 Laboratory of Nervous System Diseases and Therapy, GIGA Neuroscience, GIGA Institute, University of Liège, Liège, Belgium; 2 Department of Neurosurgery, CHU Liège, Liège, Belgium; 3 Department of Neurology, CHU Liège, Liège, Belgium; 4 Department of Pediatrics, Division of Hematology-Oncology, CHU Liège, Liège, Belgium

**Keywords:** Children, Diffuse infiltrating pontine glioma, Diffuse midline glioma, Glioblastoma, Glioma stem cells, Histone, High-grade glioma, Pediatric, Slow-cycling cells, Subventricular zone

## Abstract

Pediatric high-grade gliomas (pHGG) are brain tumors occurring in children and adolescents associated with a dismal prognosis despite existing treatments. Therapeutic failure in both adult and pHGG has been partially imputed to glioma stem cells (GSC), a subset of cancer cells endowed with stem-like cell potential and malignant, invasive, adaptative, and treatment-resistant capabilities. Whereas GSC have largely been portrayed in adult tumors, less information has been provided in pHGG. The aim of our study was to comprehensively document the stem-like capacities of seven in-use pediatric glioma cell cultures (Res259, UW479, SF188, KNS42, SF8628, HJSD-DIPG-007 and HJSD-DIPG-012) using parallel in vitro assays assessing stem cell-related protein expression, multipotency, self-renewal and proliferation/quiescence, and in vivo investigation of their tumorigenicity and invasiveness. Data obtained from in vitro experiments revealed glioma subtype-dependent expression of stem cell-related markers and varying abilities for differentiation, self-renewal, and proliferation/quiescence. Among tested cultures, *DMG H3-K27 altered* cultures displayed a particular pattern of stem-like markers expression and a higher fraction of cells with self-renewal potential. Four cultures displaying distinctive stem-like profiles were further tested for their ability to initiate tumors and invade the brain tissue in mouse orthotopic xenografts. The selected cell cultures all showed a great tumor formation capacity, but only *DMG H3-K27 altered* cells demonstrated a highly infiltrative phenotype. Interestingly, we detected *DMG H3-K27 altered* cells relocated in the subventricular zone (SVZ), which has been previously described as a neurogenic area, but also a potential niche for brain tumor cells. Finally, we observed an SVZ-induced phenotypic modulation of the glioma cells, as evidenced by their increased proliferation rate. In conclusion, this study recapitulated a systematic stem-like profiling of various pediatric glioma cell cultures and call to a deeper characterization of *DMG H3-K27 altered* cells nested in the SVZ.

**Supplementary Information:**

The online version contains supplementary material available at 10.1186/s40478-023-01586-x.

## Introduction

Pediatric high-grade gliomas (pHGG) account for approximately 15% of central nervous system (CNS) tumors occurring in children and adolescents [39] and are associated with dramatic clinical outcome for the young patient despite existing therapies [11]. pHGG represent a heterogeneous group of malignancies, with distinct molecular features and spatiotemporal patterns [32]. In the fifth edition of the WHO classification of CNS tumors, pHGG have been subdivided into different entities, designated as 1) *Diffuse midline glioma (DMG), H3-K27 altered*, 2) *Diffuse hemispheric glioma, H3 G34-mutant*, 3) *Diffuse pHGG, H3-wildtype and IDH-wildtype* and 4) *Infantile-type hemispheric gliomas* [31].

In pHGG as well as in adult glioblastoma (GBM), therapeutic failure has been attributed to tumor heterogeneity, cell infiltration through the brain, and resistance to treatment [40]. In the early 2000’s has been introduced the concept of glioma stem cells (GSC), a cancer cell sub-population with stem cell potential that was assessed based on cell self-renewal, balance in quiescence to high proliferation, multipotency and tumor initiation. These properties confer tumorigenic, invasive, adaptative, and treatment-resistant capabilities to GSC [14, 43].

The subventricular zone (SVZ), edging the walls of the lateral ventricles, is the major persistent neurogenic niche in the adult human and mouse brain [46]. In both adult GBM and pHGG, tumors that are in direct contact with the SVZ have been associated with a poor prognosis [27, 29, 35, 36], suggesting there could be important implications of the SVZ environment on glioma cell maintenance. Preclinical data from our lab provided evidence that SVZ-nested adult GBM cells show improved tumor-initiating capacities [28] and become resistant to ionizing radiation [19], features that were associated to a more pronounced “stem-like” phenotype, i.e. GSC. In pHGG, especially *DMG H3-K27 altered*, a similar invasion of the SVZ has been proposed [44]. However, the presence of GSC in the pediatric SVZ remains unknown.

In this work, we aimed to shed light on the “stem-like” properties of pHGG cultures that have been used in the neuro-oncology research field for their genetics, molecular subtypes, and drug resistance mechanisms. We examined the stem-like features of these cell cultures, using in vitro functional tests assessing stem cell-related protein expression, multipotency, self-renewal and proliferation/quiescence. Based on the results, cultures with distinct “stem-like” profiles were further selected for mouse orthotopic xenografts, in which we investigated the relative infiltration of the SVZ and subsequent phenotypic modulation. Altogether, our study adds to the growing body of literature by providing a systematic stem-like profiling of various pHGG cell cultures, to help shed light on novel treatment approaches for these dreadful tumors.

## Material and methods

### Cell culture

The human pediatric cell lines Res259, UW479, SF188, KNS42, HSJD-DIPG-007 and HSJD-DIPG-012 were kindly provided by Dr. Samuel Meignan (from Oscart Lambret Center, Lille, France). SF8628 were purchased from Sigma-Aldrich (SCC127). All cell lines were cultured according to the provider’s instructions. Res259, UW479, SF188, KNS42 and SF8628 cells were cultivated as adherent monolayers in Dulbecco’s modified Eagle’s medium and Nutrient Mixture F-12 (DMEM/F12) containing 10% fetal bovine serum (FBS, Invitrogen) and 1% penicillin/streptomycin (ThermoFisher Scientific). HSJD-DIPG-007 and HSJD-DIPG-012 were cultured as neurospheres in neural precursor cell (NPC) medium consisting in DMEM/F12 serum-free medium containing 2% of B27 without vitamin A (Life Technologies) and supplemented with recombinant epidermal growth factor (EGF, 20 ng/mL, Peprotech), fibroblast growth factor 2 (FGF-2, 10 ng/mL, Peprotech), and 1% penicillin/streptomycin (ThermoFisher Scientific). Prior to intracerebral implantation, Res259, KNS42, HSJD-DIPG-007 and HSJD-DIPG-012 cells were transduced with Lenti6-CMV-RFP-Luc (MOI 100) for stable expression of luciferase and red fluorescent protein (RFP), with the help of the GIGA Viral Vectors platform from Liège University. All cultures were maintained at 37 °C under humidified atmosphere containing 5% carbon dioxide.

### Next-generation DNA sequencing

Cell cultures were profiled using a custom SeqCap EZ HyperCap hybridization-based capture panel protocol (Roche Sequencing and Life Science Kapa Biosystems) targeting protein-coding exons as well as specific targets areas (promotor or intronic regions) of 95 cancer-associated genes, as previously described [6]. Briefly, genomic DNA was enzymatically fragmented, libraries were prepared using the KAPA HyperPlus Library Preparation Kit (Roche Sequencing) and captured using biotinylated XGen lockdown probes (Integrated DNA Technologies). Pooled libraries containing captured DNA fragments were subsequently sequenced on an Illumina NovaSeq instrument as 2 × 150 bp paired-end reads with a minimum read depth of at least × 350 coverage. The paired-end reads were mapped against the reference genome build 19 (GRCh37). Variant calling was performed using an in-house developed bio-informatics pipeline incorporating BWA for alignment, GATK for variant calling (× 2 Unified Genotyper), and Annovar for variant annotation. A third caller, Mutect 2 (GATK4) was added to increase accuracy for detecting large indels (version 11_19). It ends with a newly developed variant filtration and prioritization step that merges the annotated variant sets. The complete list of 95 genes that were sequenced is found in Additional file 2: Table S1.

### Flow cytometry

For this experiment, single-cell suspensions of 1 × 10^6^ cells cultivated in adherence (Res259, UW479, SF188, KNS42 and SF8628) or in neurospheres (HSJD-DIPG-007 and HSJD-DIPG-012) were used. For membrane staining, cells were incubated at 4 °C for 1 h in the dark with anti-human CD15-FITC, CD44-BUV737, CD49f-BV421, and CD133-PE. For intracellular staining, cells were first fixed and permeabilized (Transcription Factor Buffer Set, BD biosciences, 562,574) then cells were incubated at 4 °C for 1 h in the dark with anti-human Bmi1-Alexa Fluor647, Nestin-V450 and Sox2-PE. Similar staining was performed with isotype-matched control antibodies (BD biosciences). Antibodies are listed in the Additional file 3: Table S2. After incubation, cells were washed three times in wash buffer and stained with 7-AAD (for membrane staining) or Fixable Viability Stain 780 (FVS780) (for intracellular staining) and incubated 10 min at room temperature in the dark. After three washes with FACS buffer, samples were immediately recorded on a flow cytometer (BD Fortessa). Analysis was performed using the FlowJo software (TreeStar, Inc.). Dot plots for protein expression were generated after excluding debris and doublets by forward- and side-scatter gating and 7-AAD^+^ or FVS780^+^ cells for leaving out dead cells.

### Differentiation assay

Differentiation of cancer cells was performed by using a neural stem cell differentiation protocol. Briefly, 2 × 10^4^ cells from neurospheres (HSJD-DIPG-007 and HSJD-DIPG-012) or 2,000 adherent cells (Res259, UW479, SF188, KNS42 and SF8628) were plated in 24 wells plate on 1 × poly-L-ornithine-coated coverslips. Cells were cultured in a medium composed of 1 × Neurobasal medium (ThermoFisher Scientific), 2% B-27 Serum-Free Supplement (containing Vitamin A; ThermoFisher Scientific), 1% of Pen/Strep, 1% FBS and 2 mM GlutaMAX-I Supplement (ThermoFisher Scientific). Cells were also cultured in adherence in DMEM/F12 with 1% FBS, for spontaneous differentiation assay. Cells were incubated for 12 days at 37 °C in a humidified atmosphere of 5% CO_2_. After 12 days in culture, cells were formalin-fixed and stained for βIII-tubulin.

### Limiting dilution assay

All cell cultures were transferred into 3D neurospheres culture conditions prior to this assay. Single-cell suspensions from 3D neurospheres of each cell culture were plated into 96-well plates in NPC medium, with various seeding densities (0, 1, 2, 4, 8, 16, 32, 64, 128 and 256 cells per well). After cell seeding, the plates were incubated at 37 °C for 7 days. On day 7, each well was observed under a 10 × magnification for the determination of individual tumor cell spheres formation (> 40 µm). “Positive” wells (containing spheres) were counted, log fraction (vs wells w/o spheres) was plotted, and data were analyzed using the extreme Limiting Dilution Analysis (http://bioinf.wehi.edu.au/software/elda/index.html).

### Proliferation/quiescence assays

Cell cycling rate was assessed using CellTrace Violet cell proliferation kit (Invitrogen). Briefly, single-cell suspensions from adherent (Res259, UW479, SF188, KNS42 and SF8628) or neurospheres (HSJD-DIPG-007 and HSJD-DIPG-012) cultures were incubated with 5 μM CellTrace for 20 min at 37 °C, washed, and grown for 1–5 days. Fluorescence intensity was measured at the indicated time points by flow cytometry (FACSCanto, Becton Dickinson) and analyzed using FlowJo software (TreeStar, Inc.). Label Retaining Cell (LRC) population was defined as the cell population remaining above the threshold of fluorescence determined at day 0.

Cell proliferation was evaluated using a 5-ethynyl-2ʹ-deoxyuridine (EdU) incorporation assay (Click-iT EdU Assays) (Roche Applied Science, Indianapolis, IN, USA). 5 × 10^3^ cells of each cell line were seeded in 96-well plates and cultured overnight in serum-free medium. After incubation, medium was replaced by media with 10% FBS. Then, EdU was added to culture medium at a final concentration of 10 µM and incubated for 3 h (a “No EdU” negative control was done at this step). After incubation, 50 µL/well of staining solution was added and cells were incubated for 20 min at room temperature, protected from light. After staining, cells were washed with phosphate buffered saline (PBS) with triton 0.1% (3 × 5 min) and counterstained with DAPI for 5 min at room temperature. Cells were imaged and cell proliferation rate was analyzed and expressed as EdU positive cells relative to the total cell number. All experiments were performed in three independent sessions.

### Intracranial glioma cell transplantation

Female *nu/nu* immunodeficient mice (Crl:NU-*Foxn1*^*nu*^) (P40) were obtained from Charles River Laboratories (Wilmington, UK). Mice were anesthetized with an intraperitoneal injection of a Rompun (Sedativum 2%, Bayer, Bruxelles, Belgium) and Ketalar (Ketamin 50 mg/mL, Pfizer, Bruxelles, Belgium) solution (V/V) freshly prepared. The mouse head was restrained within a stereotactic frame, allowing a precise and reproducible injection site. Using the bregma as a landmark for the coordinates, Res259 and KNS42 cells (cultivated in adherence) were injected in the right striatum (− 0.5 mm AP, + 2.5 mm VL, + 2 mm DV) and HSJD-DIPG-007 and HSJD-DIPG-012 cells (cultivated in neurospheres) were injected in the pons (+ 5 mm AP, + 0.5 mm VL, + 5 mm DV), as a suspension of 50,000 (Res259 and KNS42) or 100,000 (HSJD-DIPG-007 and HSJD-DIPG-012) cells in 2 μL of sterile PBS. Monitoring of tumor growth was performed with in vivo bioluminescence imaging. Mice were sacrificed from the first signs of apparent discomfort (e.g. immobility, significant weight loss, absence of reaction) arising at 6 weeks after injection for KNS42 and Res259 cells, and around 8 weeks for HSJD-DIPG-007 or HSJD-DIPG-012.

### Brain tissue processing

Mice were euthanized with a Euthasol vet. injection (Sodium pentobarbital 400 mg/mL, Produlab Pharma B.V, Forellenweg, Netherlands) before intracardiac perfusion with ice-cold NaCl 0.9% solution (VWR International, Prolabo, USA) containing heparin (5000 i.u./ml, Leo Pharma) followed by paraformaldehyde (PFA) 4% in PBS. Brains were removed and postfixed in PFA 4% for 24 h. Prior to tissue cryosectioning, brains were cryoprotected for 24 h in PBS containing 30% sucrose before being flash frozen at − 80 °C. Fourteen micrometers thick coronal (Res259 and KNS42) or sagittal (HSJD-DIPG-007 and HSJD-DIPG-012) sections were cut on a cryostat and stored at − 20 °C.

Prior to light-sheet microscopy, PFA-fixed brains were clarified as previously described [47]. Briefly, half-brains were first infused for 24 h with a 4 °C hydrogel cocktail of acrylamide, bisacrylamide monomers, formaldehyde and thermally triggered initiators. Hydrogel polymerization was induced at 37 °C during 3 h, followed by the extraction and wash of the brain tissue in borate-buffered 4% sodium-dodecyl-sulfate (SDS) for 24 h at 37 °C. Half-brains were then clarified using X-Clarity Tissue Clearing System II in an Electrophoretic Tissue Clearing Solution during 24 h at room temperature. The resulting lipid-extracted and structurally stable tissue–hydrogel hybrids were washed in PBS with 0.1% of Triton X-100, for 2 days at room temperature, and finally stored in PBS-azide at 4 °C. Finally, half-brains were immersed in a refractive index (RI) homogenization solution (RIMS or Refractive Index Matching Solution; RI ~ 1.460) to render it transparent to light.

### Immunostaining and image acquisition

Cells or brain sections were permeabilized with respectively 0.1% or 0.2% Triton X-100 PBS solution, and unspecific binding sites were blocked using PBS with 10% donkey serum. Brain sections or cells were incubated with primary antibodies (Additional file 4: Table S3) diluted in PBS containing 0.1% donkey serum, followed by a second incubation with conjugated secondary antibodies (1:500, Jackson ImmunoResearch Laboratories). Cells or brains image acquisition were performed respectively with epifluorescence microscope Zeiss Apotome and Zeiss Axioscan 7.

2D and 3D images of clarified half-brains were taken with a dual illumination lightsheet Z1 (Zeiss) fluorescence microscope. Images were stitched and reconstructed using Arivis Vision 4D software. For detection of the RFP signal, images were finally analyzed using Imaris (version 9.0) software and referenced using Allen Mouse Brain Atlas.

### Statistical analysis

Data were processed and analyzed with the GraphPad Prism 8 software. Results are reported as median ± range (min/max) and analyzed using Kruskall-Wallis comparative analyses, with the n described as the number of biological samples or independent experiments. Statistical significance was set at *p* < 0.05.

## Results

### Molecular characterization of seven cell cultures derived from low- to high-grade pediatric gliomas

**Fig. 1:**
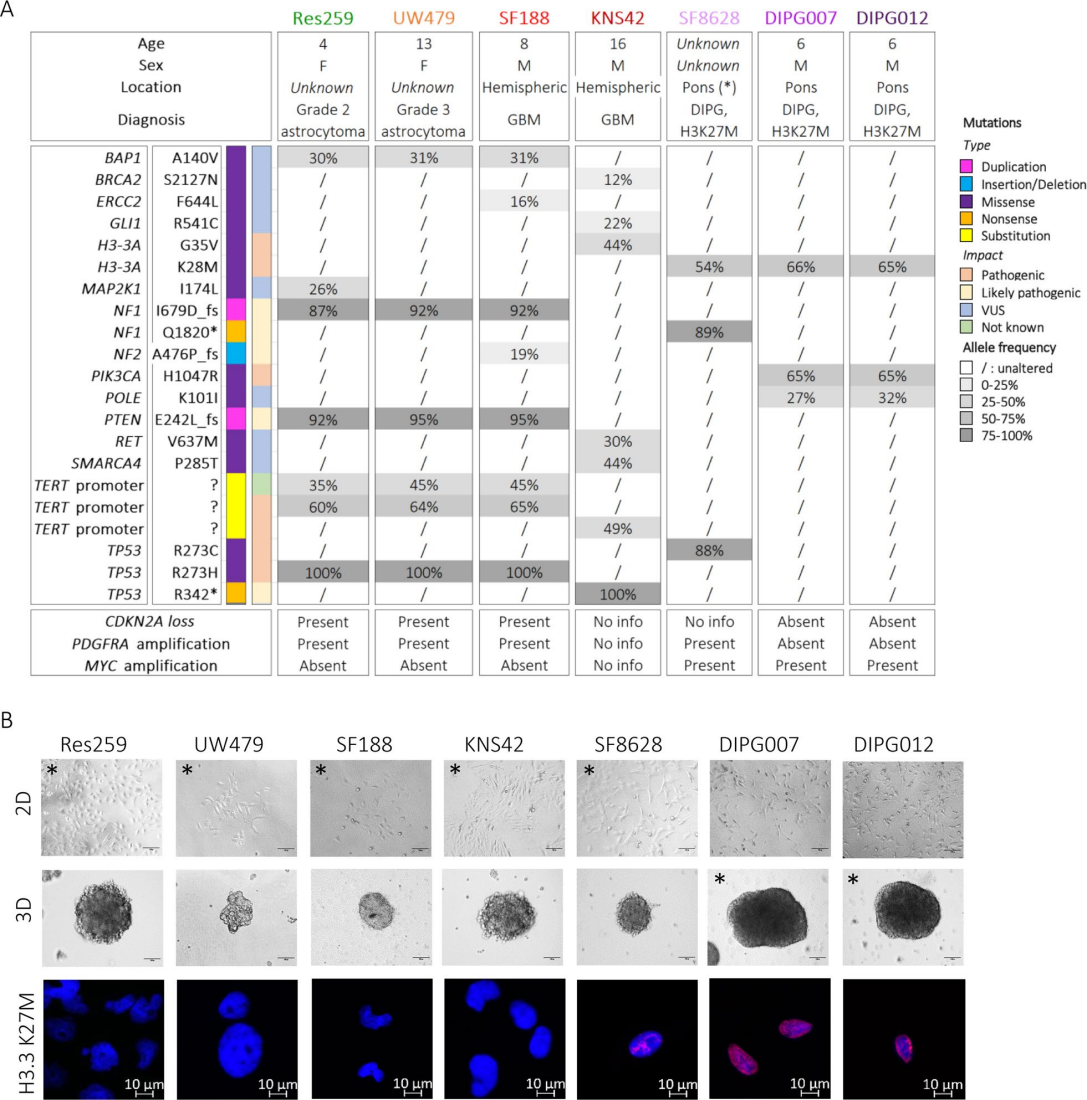
Morphological and molecular characterization of seven pediatric-type glioma cell cultures. **A** Targeted DNA sequencing of seven pediatric glioma cultures (Res259, UW479, SF188, KNS42, SF8628, HSJD-DIPG-007, HSJD-DIPG-012) was performed using SeqCap technology. 95 cancer-associated genes were studied. Detected mutations (and the resulting protein modification) are indicated according to their type and pathological impact. The allele frequency is indicated for each of the detected mutations. (*) presumed location based on DIPG diagnosis. **B** Phase-contrast pictures of the seven pediatric glioma cultures in two-dimensional (2D) and three-dimensional (3D) culture conditions. Asterisks represent initial/recommended culture conditions of the respective cell cultures (Scale bars = 100 µm). Presence of the H3.3 K27M mutation was confirmed via immunocytofluorescence (Scale bar = 10 µm). Abbreviations: *F* female, *GBM* glioblastoma, *DIPG* diffuse intrinsic pontine glioma, *M* male, *VUS* variant of uncertain significance

We started this study with seven cell cultures isolated from low-grade (Res259) to high-grade (UW479, SF188, KNS42, SF8628, HJSD-DIPG-007 and HJSD-DIPG-012) pediatric gliomas. Using next-generation DNA sequencing, we profiled 95 cancer-related genes in these cell cultures (Fig. 1A). Based on these results, Res259, UW479 and SF188 could not be associated to any pHGG entity. As expected from the parental tumor diagnosis, SF8628, HJSD-DIPG-007 and HJSD-DIPG-012 were characterized by a H3.3-K27 mutation (H3.3-K27M), confirmed by immunofluorescence (Fig. 1B) and corresponded to pontine *DMG, H3-K27 altered*, formerly called diffuse infiltrative pontine gliomas (DIPG). We confirmed the *Diffuse hemispheric glioma, H3 G34-mutant* subgroup for KNS42, as previously described [7].

From their initiation, all cultures were either grown as adherent monolayers or tridimensional tumor spheres depending on their initial culture setup, but all cell types could be cultured in both experimental conditions (Fig. 1B).

### Expression of stem-cell associated surface and intracellular markers distinguishes subgroups among pediatric glioma cell cultures

**Fig. 2:**
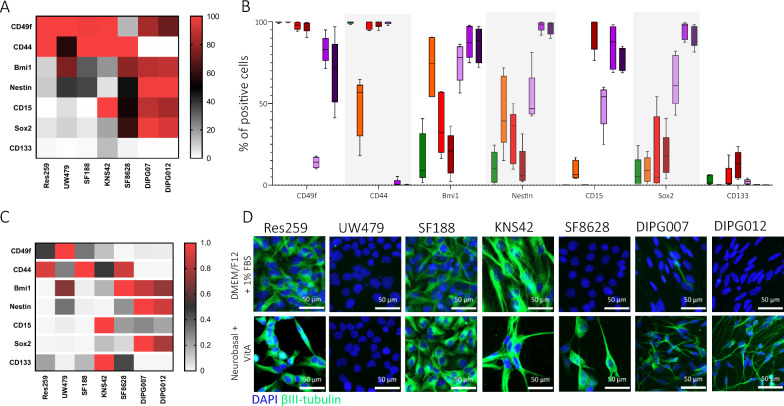
Expression of stem cell-associated proteins and evaluation of multipotency in pediatric-type glioma cell cultures. **A** Heat map with continuous color gradient representing the median percentage of cells expressing given markers in each cell culture. **B** Histogram representing the percentage of cells expressing the stem cell-associated markers CD49f, CD44, Bmi1, Nestin, CD15, Sox2 and CD133 for each cell culture (results are represented as median with range min/max, bow represents P25 and P75, N = 5 independent experiments). Cells were cultivated in adherence (Res259, UW479, SF188, KNS42 and SF8628) or in neurospheres (HSJD-DIPG-007 and HSJD-DIPG-012). **C** Signal intensity was normalized for each marker, among the seven cell pHGG cultures (0 = lowest value, 1 = highest value; median values calculated on N = 5 experiments). **D** Representative pictures of the βIII-tubulin expression (green) after differentiation towards neuronal lineage, in adherence in DMEM/F12 + 10% FBS or under neuronal induction medium for 12 days (Scale bar = 50 µm)

Many proteins have been suggested as playing an essential role in GSC behavior, especially in self-renewal, proliferation, quiescence and tumorigenicity [14] and therefore have been proposed as putative GSC-specific identification markers. Based on the literature, we selected a panel of seven membrane (i.e. CD49f, CD44, CD15, CD133), cytoplasmic (i.e. Nestin) and nuclear (i.e. Bmi1, Sox2) proteins [1, 17]. We assessed the percentage of positive cells for each protein of the panel via flow cytometry. *DMG H3 K27-altered* derived cell cultures (i.e. SF8628, HSJD-DIPG-007 and HSJD-DIPG-012) presented a distinguishable protein expression profile, compared to other pediatric glioma cultures (Fig. 2A–C). In this subtype, highly represented proteins included Bmi1 (77.9 to 88.6% of positive cells), Nestin (48.6 to 97.8% of positive cells), CD15 (54.2 to 87.7% of positive cells) and Sox2 (67.0 to 90.5% of positive cells). Moreover, H3-K27M cells had the lowest CD49f positive cell population (from 14.1 to 83.1% of positive cells) compared to non H3K27M cells (98.0 to 100% of positive cells). In opposite, HSJD-DIPG-007 and HSJD-DIPG-012 did not show any expression of CD44, whereas Res259, UW479, KNS42, SF188 were highly positive for this marker (56.9 to 99.8% of positive cells). CD44 expression in SF8628 was high compared to the two other *DMG H3K27-altered* cell cultures. Finally, KNS42 cells expressed all the seven proteins of the panel, and was the only culture that showed remarkable expression of CD133 (13.1% of positive cells) (Fig. 2A–C).

### Neuronal-like differentiation can be observed in most pediatric glioma cell cultures

During development but also in particular adult tissue niches, normal stem cells differentiate into functional cells from various lineages. In cancer tissues including glioma, cancer stem cells (CSC) also have been described as endowed with multipotency [48, 50]. To induce a neuronal differentiation process in pediatric glioma cells, we cultivated them for 12 days in adherence on glass coverslips, either in 1% FBS medium, or in neuronal induction medium. Then, the cells were fixed and immunostained for βIII-tubulin. Res259, SF188 and KNS42 demonstrated a spontaneous βIII-tubulin expression after 12 days in adherent culture. Similar βIII-tubulin expression was observed in neuronal differentiation medium. This neuronal differentiation medium specifically induced βIII-tubulin expression in the *DMG H3K27-altered* derived cells SF8628, HSJD-DIPG-007 and HSJD-DIPG-012. In our hands, UW479 did not express βIII-tubulin in any of the tested culture conditions (Fig. 2D).

### Pediatric glioma cell cultures exhibit variable self-renewal potential

**Fig. 3:**
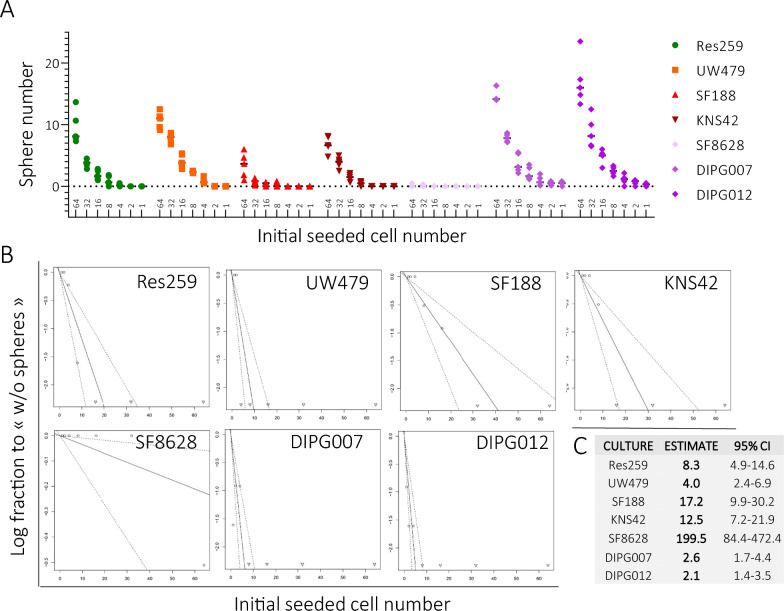
Evaluation of the self-renewal of the seven pediatric-type glioma cell cultures by limiting dilution assay. **A** Quantification of the number of spheres at day 7 in each pediatric glioma cell culture, depending on the number of cell(s) seeded. Data are represented by median and range min/max (N = 5 independent experiments) **B** Log-fraction plots of the limiting dilution assay fitted to the data encoded in ELDA software (“dose”, “tested”, “response” for each culture). “Dose” data refers to the number of seeded cells (i.e. 128, 64, 32, 16, 8, 4, 2, 1). “Tested” referred to the number of independent experiments (i.e. N = 5). A “response” was considered positive when the 3 replicate wells for one dose included in average ≥ 3 neurospheres (diameter ≥ 40 µm). The slope represents the log “active cell fraction”. Dotted lines delimit the 95% confidence interval. The data values with zero negative response at each dose is represented by a down-pointing triangle. **C** Results of the limiting dilution assay expressed as the estimated number of cells among which a cell with self-renewal ability is counted (= 1/stem cell frequency) (with 95% CI)

In an attempt to evaluate the stem-like properties of each of these glioma cultures, we submitted them to various functional assays. It is accepted that GSC grown as neurospheres exhibit similar characteristics to physiological neural stem cells also grown as neurospheres, including the ability to self-renew [38]. To test whether the pediatric glioma cell cultures were self-renewable, we employed a limiting dilution assay (LDA) [23], in which we seeded cells from 256 to 1 cell per well, before culturing them for 7 days. All the cell cultures were able to generate neurospheres from at least 64 cells with an average number of neurospheres of 9.5 (SD:2.6) for Res259, 10.7 (SD: 1.3) for UW479, 3.4 (SD: 2.0) for SF188, 6.8 (SD: 1.3) for KNS42, 0.2 (SD: 0.2) for SF8628, 14.9 (SD: 1.2) for HSJD-DIPG-007 and 16.9 (SD: 3.9) for HSJD-DIPG-012. Only HSJD-DIPG-007 and HSJD-DIPG-012 were able to form sphere starting from 1 cell per well (Fig. 3A and Additional file 1: Figure S1). The number of wells containing spheres (across 5 independent experiments) was entered in the ELDA calculation tool [24] and the results showed the following proportion of cells with self-renewing ability in each cell cultures: Res259 12% (1/8.3; CI: 1/4.9–1/14.6), UW479 25% (1/4.0; CI: 2.4–6.9), SF188 6% (1/17.2; CI: 9.9–30.2), KNS42 8% (1/12.5; CI: 7.2–21.9), SF8628 0.5% (1/199.5; CI: 84.4–472.4), HSJD-DIPG-007 38% (1/2.6; CI: 1.7–4.4) and HSJD-DIPG-012 48% (1/2.1; CI: 1.4–3.5) (Fig. 3B, C). Overall test for differences in self-renewing cell frequencies between any of the groups was significant (Chisq: 184; *p* = 6.05.10^–37^). These results suggest that the *DMG H3-K27-altered* derived cultures HSJD-DIPG-007 and HSJD-DIPG-012 contain the highest number of auto-renewable, stem-like cells. In opposition, SF188, KNS42 and SF8628 supposedly contain a minority (< 10%) of these self-renewable cells.

### Pediatric glioma cell cultures are differentially enriched in proliferative vs quiescent cells

**Fig. 4:**
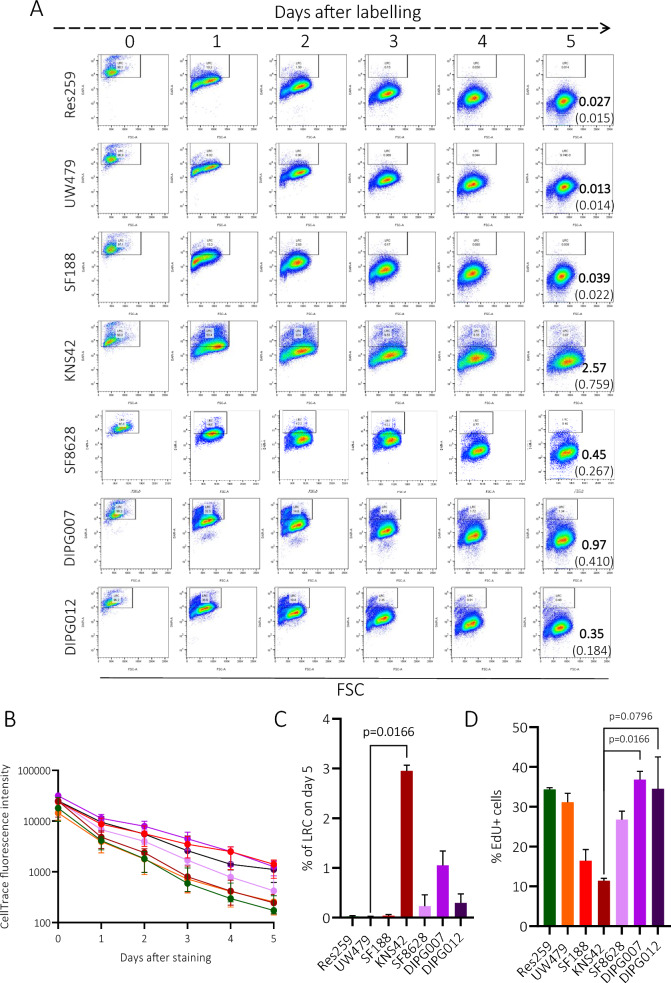
Study of the quiescence/proliferation in pediatric-type glioma cell cultures by Cell Trace and EdU incorporation assay. **A** Representative dot plots highlighting label-retaining cells (LRC) (gate based on day 0) each day after Cell Trace labeling. **B** Mean fluorescent intensity of the CellTrace™ labelling decreases with time in all tested cultures. **C** Numbers indicate percentage of cells that are considered as quiescent, non-dividing, detected on day 5 in culture. Summary histograms of the percentage of quiescent, non-dividing cells detected on day 5 after CellTrace™ labeling. **D** Numbers indicate percentage of proliferating, EdU-positive cells for each cell culture. Data are represented by median and range min/max, and were analyzed with Kruskall-Wallis tests (N = 5 independent experiments). Cells were cultivated in adherence (Res259, UW479, SF188, KNS42 and SF8628) or in neurospheres (HSJD-DIPG-007 and HSJD-DIPG-012)

Proliferation and quiescence represent two critical cellular states in normal stem cells [2, 37] but also in CSC, in which quiescence has been long considered as a stem-like phenotype involved in tumor propagation and resistance to treatment [2, 3, 13, 25, 42, 49]. To observe and quantify the non-dividing, quiescent cells, we employed a CellTrace labelling assay. We labelled all cells at day 0 and daily measured the percentage of LRC over 5 days [5]. The analysis of LRC on day 5 after cell trace labeling revealed the following proportions of LRC in each culture: Res259 0.027% (SD: 0.015), UW479 0.013% (SD:0.014), SF188 0.039% (SD: 0.022), KNS42 2.57% (SD: 0.759), SF8628 0.45% (SD: 0.267), HSJD-DIPG-007 0.97% (SD: 0.410), HSJD-DIPG-012 0.35% (SD: 0.184). KNS42 had a significant higher LRC population on day 5 after labeling than UW479 (*p* = 0.0166) (Fig. 4A–C). For three selected cell cultures, we applied the CellTrace labelling assay in both 2D and 3D culture conditions, and showed that the proliferation rate did not seem to be influenced (Additional file 1: Figure S2, A-B). In parallel, we also used an EdU incorporation assay for tracking the replicating cell population. Counting of EdU-positive cells in each pediatric glioma cell culture revealed various amounts of proliferative cells: Res259 31.72% (SD: 4.994), UW479 31.68% (SD: 1.524), SF188 16.24% (SD: 3.134), SF8628 26.77% (SD: 2.110), HSJD-DIPG-007 37.40% (SD: 1.308), HSJD-DIPG-012 36.73% (SD: 5.036). KNS42 had a notably lower number of proliferating cells compared to others (KNS42 11.60% (SD: 0.352)) (Fig. 4D and Additional file 1: Figure S2, C-D).

### Pediatric glioma cell cultures exhibit different growth patterns upon orthotopic implantation

**Fig. 5:**
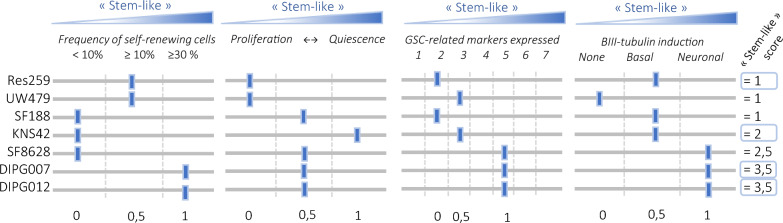
Stem-like score of the pediatric-type glioma cell cultures. Integration of the data provided using the four in vitro functional assays, and establishment of a “stem-like” score for pediatric glioma cell cultures

**Fig. 6:**
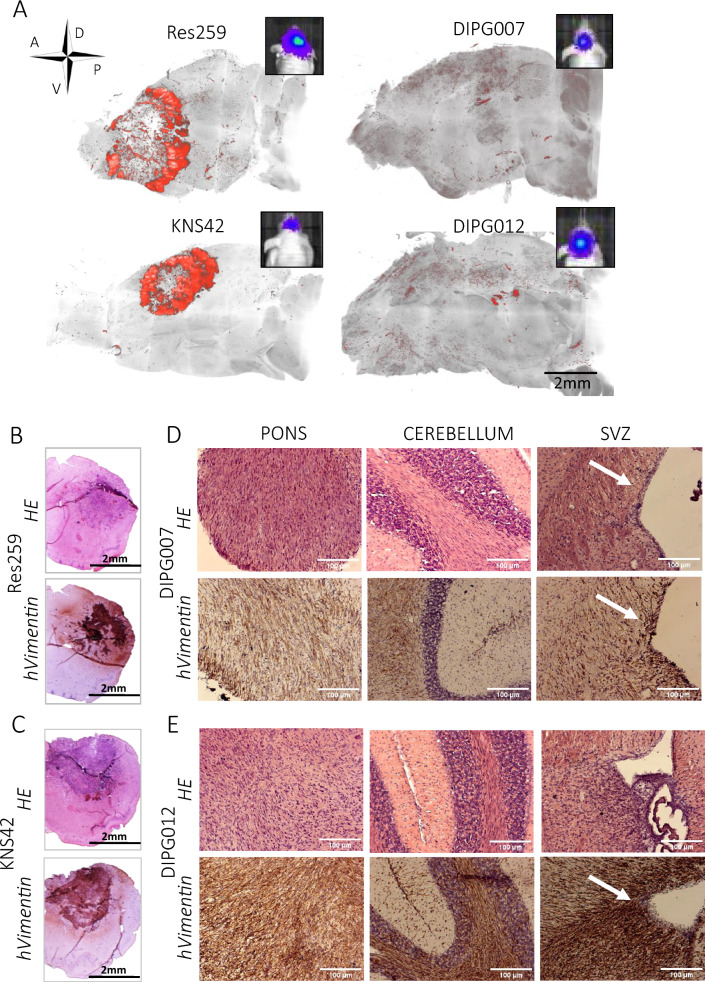
Study of Res259, KNS42, HSJD-DIPG-007 and HSJD-DIPG-012 tumor growth profiles upon orthotopic xenografts. **A** 3D representation of the RFP + (red) tumor cells in the brain right hemisphere after tissue clarification (with examples pictures of tumor bioluminescent signal at endpoint). **B**–**E** Sagittal or coronal mice brain sections were stained with hematoxylin & eosin staining and anti-human vimentin. The whole right hemisphere or higher magnification pictures from the pons, the cerebellum and the subventricular zone (SVZ) (pointed by white arrow) are shown for **B** Res259, **C** KNS42, **D** HSJD-DIPG-007 and **E** HSJD-DIPG-012. Cells were cultivated in adherence (Res259, UW479, SF188, KNS42 and SF8628) or in neurospheres (HSJD-DIPG-007 and HSJD-DIPG-012)

The very initial definition of CSC has proposed these cells as critical drivers of tumor initiation and propagation [4]. In the present investigation of stem-like features of pediatric glioma cells, we therefore aimed to interrogate their tumorigenic potential in vivo. Based on the integration of the previous in vitro results (Fig. 5), we selected four pediatric cell cultures with different profiles, which we used in an orthotopic xenograft mouse model. Cells were transduced for stable expression of RFP and luciferase, then engrafted into athymic nude mice brains. Res259 and KNS42 were implanted in the right striatum, whereas HSJD-DIPG-007 and HSJD-DIPG-012 were implanted in the pons. Tumor growth was monitored using in vivo bioluminescence imaging, and tumor phenotype was analyzed at endpoint based on brain sections or clarified 3D brain hemispheres. The four pediatric glioma cell cultures were able to generate brain tumors in vivo (Fig. 6A and Additional file 1: Figure S3, A). Res259 and KNS42 formed large, dense and angiogenic/necrotic-like tumors around their injection site, without evident signs of cell invasion away from the tumor core (Fig. 6B, C and Additional file 1: Figure S3, B). On the opposite, HSJD-DIPG-007 and HSJD-DIPG-012 tumor cells scattered throughout the whole brain. Infiltrated single cells were hardly detected using lightsheet microscopy analysis of whole hemispheres, but histological analysis of the tissue revealed loads of cells in the pons, but also in the cerebellum and the thalamus. Interestingly, cells also infiltrated the SVZ (Fig. 6D, E).

### The SVZ influences pediatric glioma cell phenotype

**Fig. 7:**
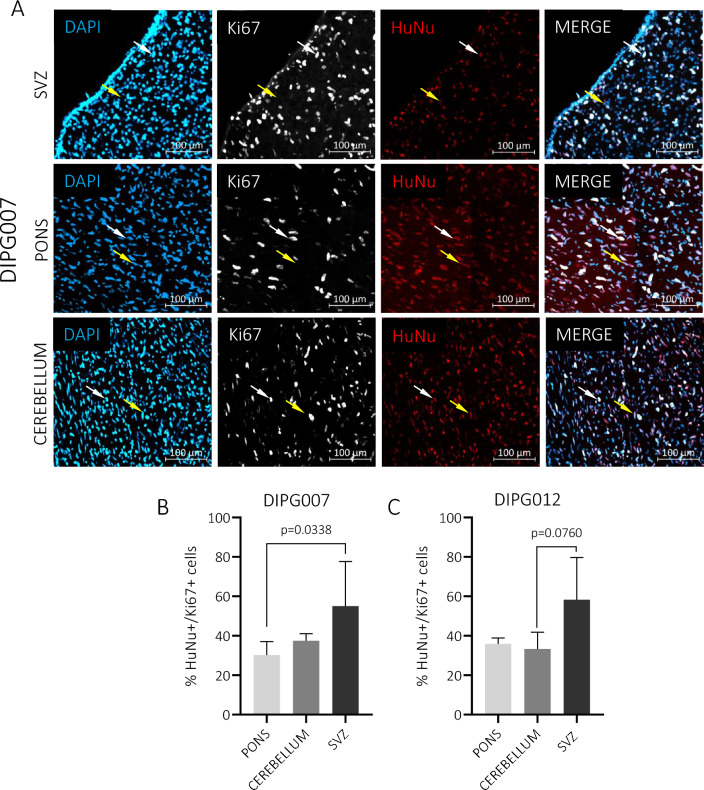
Study of HSJD-DIPG-007 and HSJD-DIPG-012 proliferation upon orthotopic xenograft. **A** Sagittal brain sections implanted with HSJD-DIPG-007 cells were immunostained for anti-human nuclei (red), Ki67 (white) and DAPI (blue) counterstaining. Representative pictures into the rostral subventricular zone (SVZ), pons and cerebellum. White arrow indicates HuNu + /Ki67 + cells, yellow arrow indicates HuNu + /Ki67 − cells. **B**, **C** Histograms of the percentages of double-positive human nuclei (HuNu +) and Ki67 (Ki67+) cells compared between the pons, cerebellum and SVZ for **B** HSJD-DIPG-007 and **C** HSJD-DIPG-012 tumors. Data are represented as median and range min/max, and were analyzed with Kruskall-Wallis tests (N = 3 independent experiments)

We observed that pediatric glioma cells, especially *DMG H3-K27-altered* derived cells highly invade the brain tissue and reach the SVZ. Interestingly, such SVZ-oriented invasion has recently been proposed for pontine DMG cells [44], and also for adult GBM cells, especially stem-like cells [18]. Additionally, our team demonstrated that adult SVZ-nested GBM cells were resistant to ionizing radiation [19]. We were therefore interested in looking at the phenotype of HSJD-DIPG-007 and HSJD-DIPG-012 that relocated to the SVZ. We observed that, in all pHGG models, tumor cells remained similarly Nestin-positive and SOX2-positive in all investigated brain areas, including the pons, cerebellum and SVZ (Additional file 1: Figure S4). We then counted the number of Ki67-positive tumor cells, which revealed a higher proportion of proliferating HSJD-DIPG-007 cells in the different parts of the SVZ compared to the pons or the cerebellum (Fig. 7A, B and Additional file 1: Figure S5, A). Similar results were obtained for HSJD-DIPG-012 (Fig. 7C and Additional file 1: Figure S5, A-B).

## Discussion

In this work, we characterized cell cultures derived from different molecular subtypes of pediatric gliomas, i.e. Res259, UW479, SF188, KNS42, SF8628, HSJD-DIPG-007 and HSJD-DIPG-012, which have been used for a number of neurooncology research studies but not always thoroughly described. We aimed at comprehensively documenting their stem-like properties and assess their tumor initiation and invasive abilities. We also investigated the impact of the SVZ on glioma cell phenotype.

The very low availability of pHGG tumor tissue derived from patients for research use worldwide has led to the preferential utilization of established pHGG cell lines, whose characteristics may obviously have deviated from those of the original tumor over time. We therefore first characterized the molecular profile of pediatric glioma cultures to observe clinically-relevant features. The latest 2021 WHO classification of tumors of the CNS individualized the HGG of pediatric type and subdivided them into four entities based on clinical, histological and molecular features [31]. Based on next-generation sequencing results, we confirmed the presence of a K27M mutation in histone H3 (H3.3-K27M) mutation in the SF8628, HJSD-DIPG-007 and HJSD-DIPG-012 cell lines, in line with the initial diagnosis of *DMG, H3-K27 altered* tumors. We also detected a H3-G34V mutation in the KNS42 cell line, as described in the literature [7, 26, 34]. However, we could not assess a tumor subgroup for the three other cell lines (Res259, UW479, SF188). It turned out that the Res259 cell culture showed phenotypic and molecular characteristics of a high-grade tumor (e.g. tumorigenicity in vivo, pathogenic mutation of TP53 in 100% of the cells) while the initial histological diagnosis of grade 2 astrocytoma [8]. These results underlie the importance of accurate histomolecular characterization retrospectively and recommend cautious data interpretation with long-term cell cultures.

We also analyzed stem cell-related protein expression, which revealed different clusters among the seven cell cultures that were studied. In particular, *DMG H3-K27 altered* derived cell lines revealed a distinct pattern, with higher expression of stem cell-related markers, e.g. Bmi1, Nestin, CD15 and Sox2, slightly lower expression of CD49f and no CD44 expression. Two of them (HSJD-DIPG-007 and HSJD-DIPG-012), grown as neurospheres, also showed the highest self-renewal ability and were able to form neurospheres from a single cell. The slightly different expression panel of SF8628 compared to the two other *H3-K27 altered DMG* cell cultures*,* and the presence of a significant CD44 expression in particular, could be related to its two-dimensional culture conditions. *H3-K27 altered DMG* cell cultures also displayed a particular ability to express the neuronal marker βIII-tubulin upon induction. Interestingly, Rakotomalala et al. [45] recently showed that the introduction of a H3.3 K27M in the originally unmutated cell lines Res259, SF188, and KNS42 induced context-dependent effects in terms of clonogenicity, transcriptomic profile, and resistance to treatment. Such results strongly suggest that the H3.3 K27M mutation governs many aspects of pHGG cell behaviors, including GSC characteristics. Altogether, these data suggest distinct stem-like features between the cells from different pHGG molecular subgroups, with *H3-K27 altered DMG* cell cultures showing a more pronounced stem-like profile. These observations are consistent with recent evidence suggesting distinct cancer stem cell models among the different HGG molecular subgroup, intermingled with putative distinct cells of origin.

In *H3-K27 altered DMG*, it is now recognized that the majority of the tumor cells are blocked in the state of oligodendrocyte precursor cells (OPC), which proliferate, self-renew and give rise to a minority of more differentiated oligodendrocyte- and astrocyte-like cells [16]. This accumulation of OPC could be related to the repressive effect of H3-K27M on the Polycomb Repressive Complex 2 (PRC2), which is required for the differentiation of OPC into oligodendrocytes. The overexpression of the PRC1 subunit Bmi1, observed in our protein expression profile and previously described [16], could represent a compensatory mechanism to the repression of PRC2 induced by H3-K27M. Additionally, it has been shown that ectopic expression of H3-K27M in OPC induces the expression of the transcription factor Sox2 [41], a regulator of embryonic and induced pluripotent stem cells [33]. Overexpression of Sox2 in H3-K27M cells was also previously reported by comparing two epigenetic (H3K27-high and H3K27 low) subpopulations identified by single-cell epigenetic analysis [20]. In the same analysis, H3-K27M expression was associated with a reduced expression of the mesenchymal-like cells marker CD44 [20], in accordance with our own observations. In the literature, other pHGG subtypes have been understudied with respect to their stemness pattern and cell of origin, probably due to difficulties in compiling large cohorts of these sub-entities. *Diffuse hemispheric glioma, H3 G34-mutant* were recently demonstrated as developing from GSX2-expressing interneuron progenitor-like cells from the SVZ [12] but, to our knowledge, data is lacking for other pHGG subtypes.

We then evaluated the propensity of four cell lines, with distinct in vitro stem-like profiles, to form tumors upon implantation and to migrate to the SVZ, and we assessed whether the SVZ could influence their phenotype. Using two orthotopic (striatum or pons) xenograft mouse models, we demonstrated the ability of pontine *DMG H3-K27 altered* cells, with stemness features, to invade the brain tissue and reach the SVZ. Of note, the two other cell cultures (i.e. Res259 and KNS42) formed large tumors that did not display evident tissue infiltration. Although widely studied in adults (for a review, see Lombard et al. [30]), relatively few data exist on the exact involvement of the SVZ in pHGG. Based on a series of autopsies, Caretti et al. observed a contact or invasion of the SVZ by pontine DMG cells in 62.5% (10/16) of the patients during the course of their disease [10]. An SVZ invasion was also radiologically identified at diagnosis in 53% (34/63) and 40% (21/52) of children and adolescents with supratentorial pHGG in two different series [27, 36]. In both studies, SVZ invasion was associated with poorer overall survival in multivariate analyses. Unfortunately, the possible impact of the mutational status H3-K27M on this association could not be robustly evaluated [27, 36]. Additionally, Qin et al. showed that pontine DMG cells, collected in the SVZ of a patient during autopsy and injected into the pons of juvenile immunodeficient mice, consistently spread into the SVZ in response to chemoattractants secreted by SVZ-hosted NPC [44]. In-depth phenotypic analysis of pHGG cells involved in these invasion patterns revealed the presence, in the infiltrating edge, of slow-cycling/quiescent cells expressing the stemness marker CD133 and adopting a mesenchymal-like morphology with a strong accumulation of N-cadherin at the cell–cell contact [2]. Interestingly, slow-cycling cells (SCC) have been observed for decades in human tumors harboring an otherwise overall fast proliferation, including adult GBM [15], where they have been linked to tumor recurrence after chemotherapy [9, 13] and invasion [21]. In pHGG, analysis of variant allele frequencies supported a model of tumor growth involving SCC that give rise to fast-proliferating progenitor-like cells and to non-dividing cells [22]. Our observations also support the existence of a SCC subpopulation coexisting with a proliferative subset in *DMG, H3-K27 altered* and *Diffuse hemispheric glioma* and *H3 G34-mutant.* They also suggest a SVZ-induced phenotypic modulation of the tumor cells, as evidenced by their increased proliferation rate in the SVZ. In different cancer types, SCC have been related to CSC and epithelial-to-mesenchymal transition-like cells. All three cancer cells harbor common traits and distinct features and could possibly describe the same population of cells in different cellular (epigenetic) states [5].

In conclusion, we presented a systematic stem-like profiling of various pediatric glioma cell cultures. We confirmed the ability of pHGG tumor cells with stem-like features to invade the SVZ and suggest the existence of phenotypic/epigenetic modulations of the tumor cells by the SVZ environment. Taken together, these observations call for a deeper understanding of the mechanisms underlying these modulations and their involvement in the whole process of tumor cell migration from the tumor mass up to the SVZ.

## Supplementary Information

**Additional file 1: Fig. S1.** Illustrative pictures of the limiting dilution assay. At day 7, starting with 256, 64, 16, 4 and 1 cell(s) per well. **Fig. S2.** Study of the quiescence/proliferation in pediatric-type glioma cell cultures by Cell Trace and EdU assays. **A** Mean fluorescent intensity of the CellTrace labelling decreases with time in Res259, HSJD-DIPG007, HSJD-DIPG012, in DMEM + 10% FBS as well as in NPC medium, in a similar rate (N = 3 independent experiments). **B** Representative phase-contrast pictures show that all three cell types are adherent in DMEM + 10% FBS, and form spheres in NPC medium. **C**, **D** Representative pictures of EdU incorporation analyzed by **C** epifluorescent microscopy (EdU-positive cells in red) and **D** via flow cytometry. White arrow indicates EdU+ cells. **Fig. S3.** Tumorigenicity of four selected pediatric-type glioma cell cultures upon orthotopic xenograft in mice. **A** Representative pictures of tumor-associated bioluminescence recorded at tumor endpoint. **B** Macroscopic view of Res259, KNS42, HSJD-DIPG-007 and HSJD-DIPG-012-engrafted right brain hemispheres after tissue clarification. **Fig. S4.** Expression of Nestin and Sox2 in *DMG K27*-altered cells after orthotopic xenografts. Sagittal brain sections of brains implanted with HSJD-DIPG-007 and HSJD-DIPG-012 were immunostained for anti-human nuclei (red), Nestin (green) or Sox2 (yellow) and DAPI (blue) counterstaining. Images are representative pictures of the subventricular zone (SVZ), pons and cerebellum. White arrow indicates Nestin+ or Sox2+ cells. **Fig. S5.** Regions of interest (ROIs) in the subventricular zone. **A** The subventricular zone (SVZ) was defined as the layer with 200 µm depth from the inside border of the lateral ventricle towards the brain parenchyma. SVZ was divided in three distinguishable regions: the rostral SVZ (ROS), the caudal SVZ (CAU) and the dorsal SVZ, below of the corpus callosum (CC). ROIs were established based on these parameters. **B** Sagittal brain sections implanted with HSJD-DIPG-012 cells were immunostained for anti-human nuclei (red), Ki67 (white) and DAPI (blue) counterstaining. Representative pictures into the rostral SVZ, pons and cerebellum. White arrow indicates HuNu+/Ki67+ cells, yellow arrow indicates HuNu+/Ki67- cells. **C**, **D** Histograms of the percentages of Ki67-positive cells in the pons, cerebellum and the different parts of the SVZ for **C** HSJD-DIPG-007 and **D** HSJD-DIPG-012 tumors.

**Additional file 2: Table S1.** List of the 95 genes sequenced by next-generation DNA sequencing.

**Additional file 3: Table S2.** Antibodies used for flow cytometry experiments.

**Additional file 4: Table S3.** Antibodies used for immunofluorescent stainings on fixed cells and brain sections.

## Data Availability

The datasets used and/or analyzed during the current study available from the corresponding author on reasonable request.

## Abbreviations

CNS: Central nervous system
CSC: Cancer stem cell
EdU: 5-Ethynyl-2ʹ-deoxyuridine
FBS: Fetal bovine serum
GBM: Glioblastoma
GSC: Glioma stem cell
LDA: Limiting dilution assay
LRC: Label retaining cell
NPC: Neural precursor cell
OPC: Oligodendrocyte precursor cells
PBS: Phosphate buffered saline
EGF: Epidermal growth factor
FGF-2: Fibroblast growth factor 2
pHGG: Pediatric high-grade glioma
RFP: Red fluorescent protein
SCC: Slow cycling cell
SVZ: Subventricular zone
