# Supplementary figures and images for: Comprehensive profiling of stem-like features in pediatric glioma cell cultures and their relation to the subventricular zone
Source: Acta Neuropathol Commun. 2023 Jun 16;11:96. doi: 10.1186/s40478-023-01586-x (PMC10276389; doi:10.1186/s40478-023-01586-x)

Figure S1

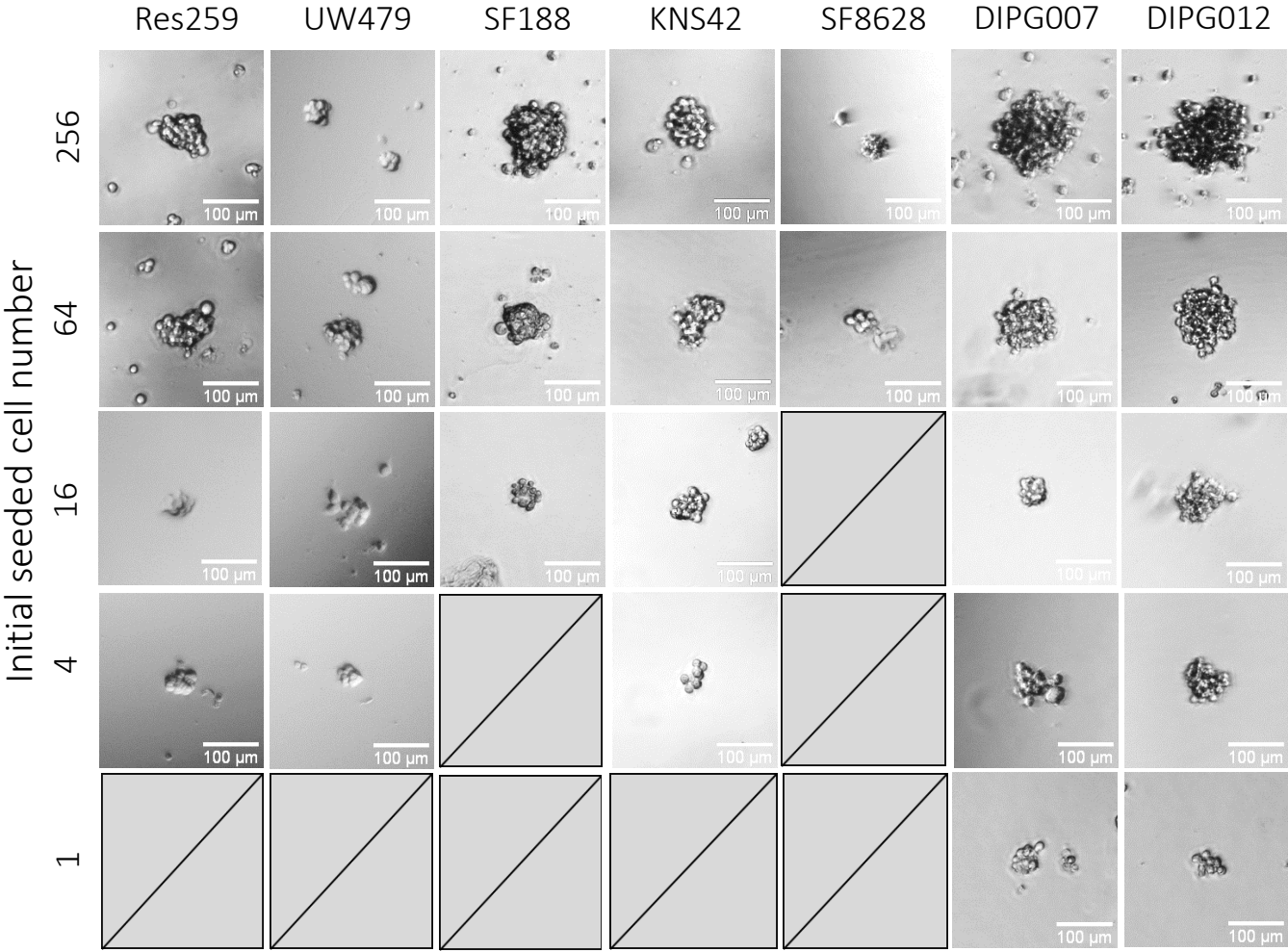

Figure S2

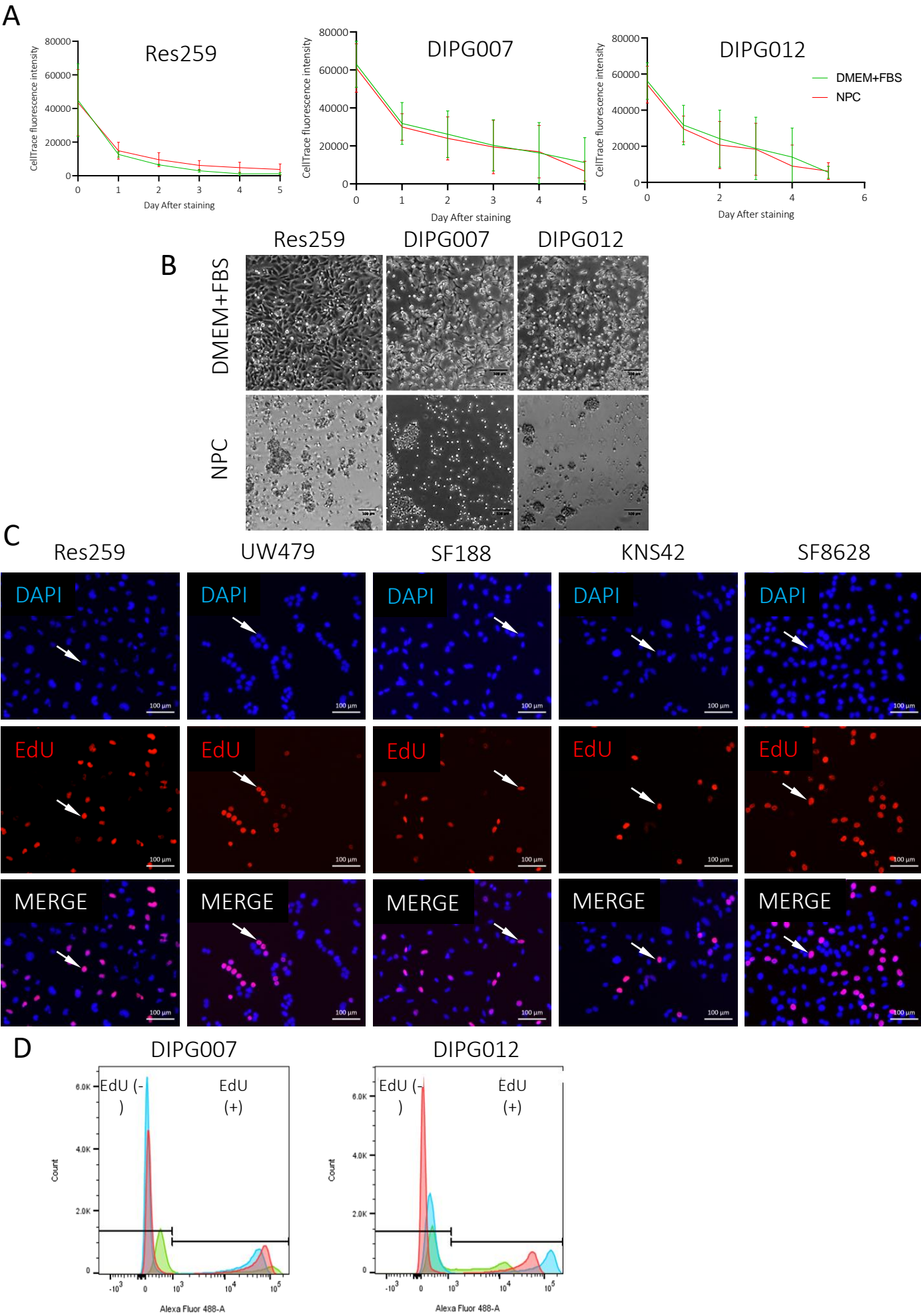

Figure S3

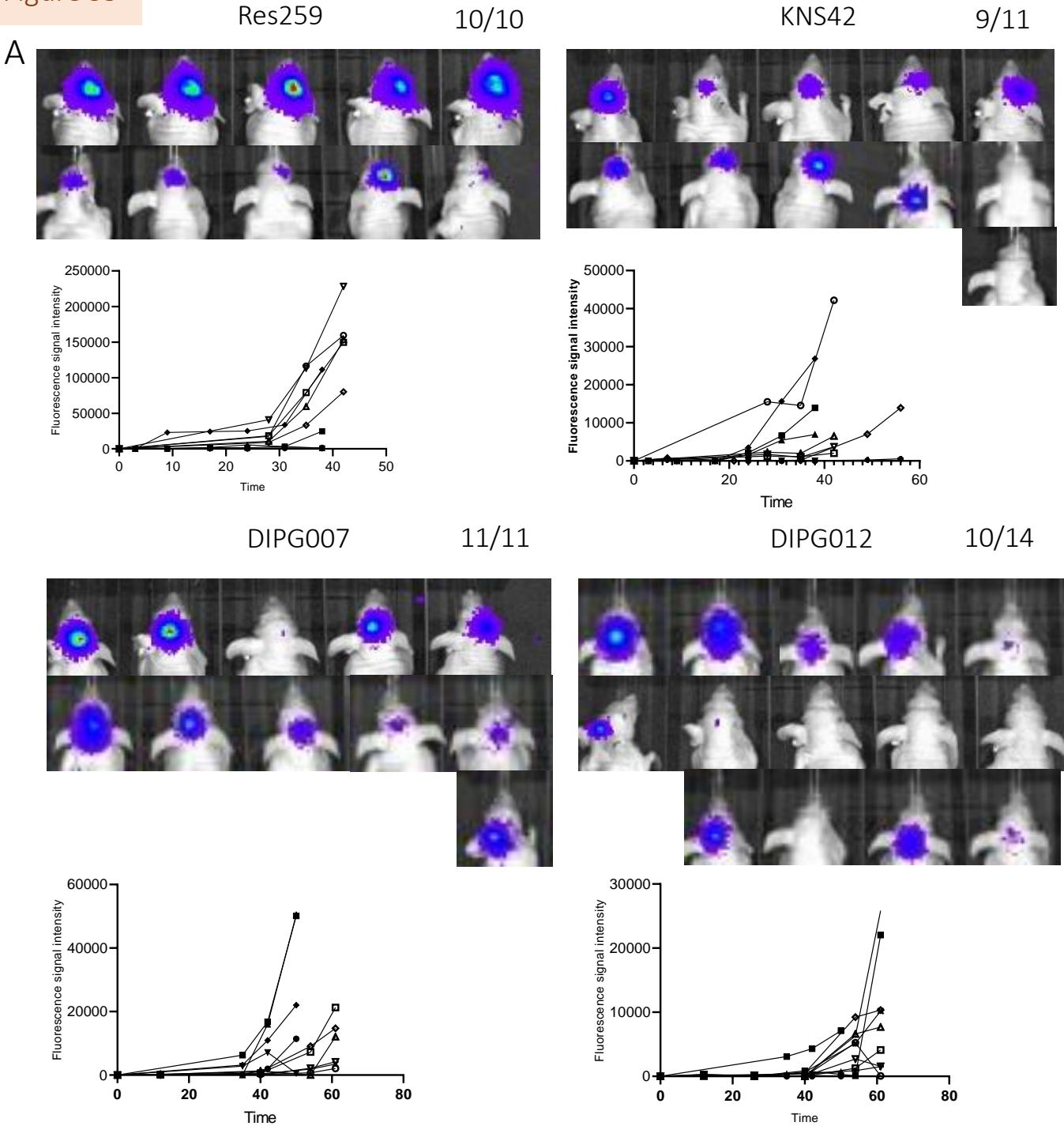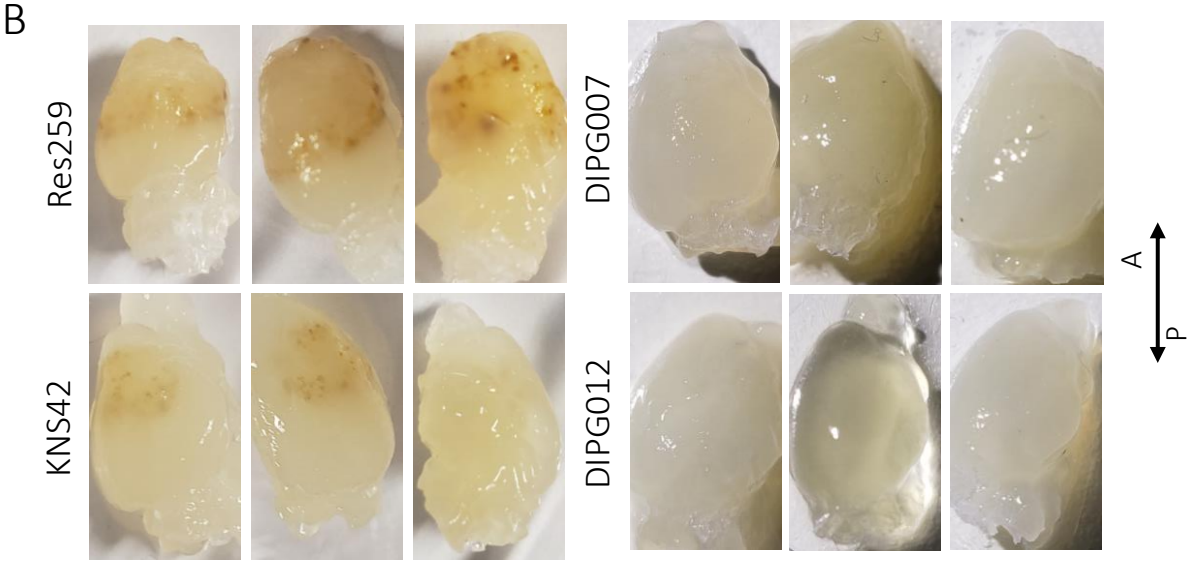

Figure S4  
(paysage)

DIPG007

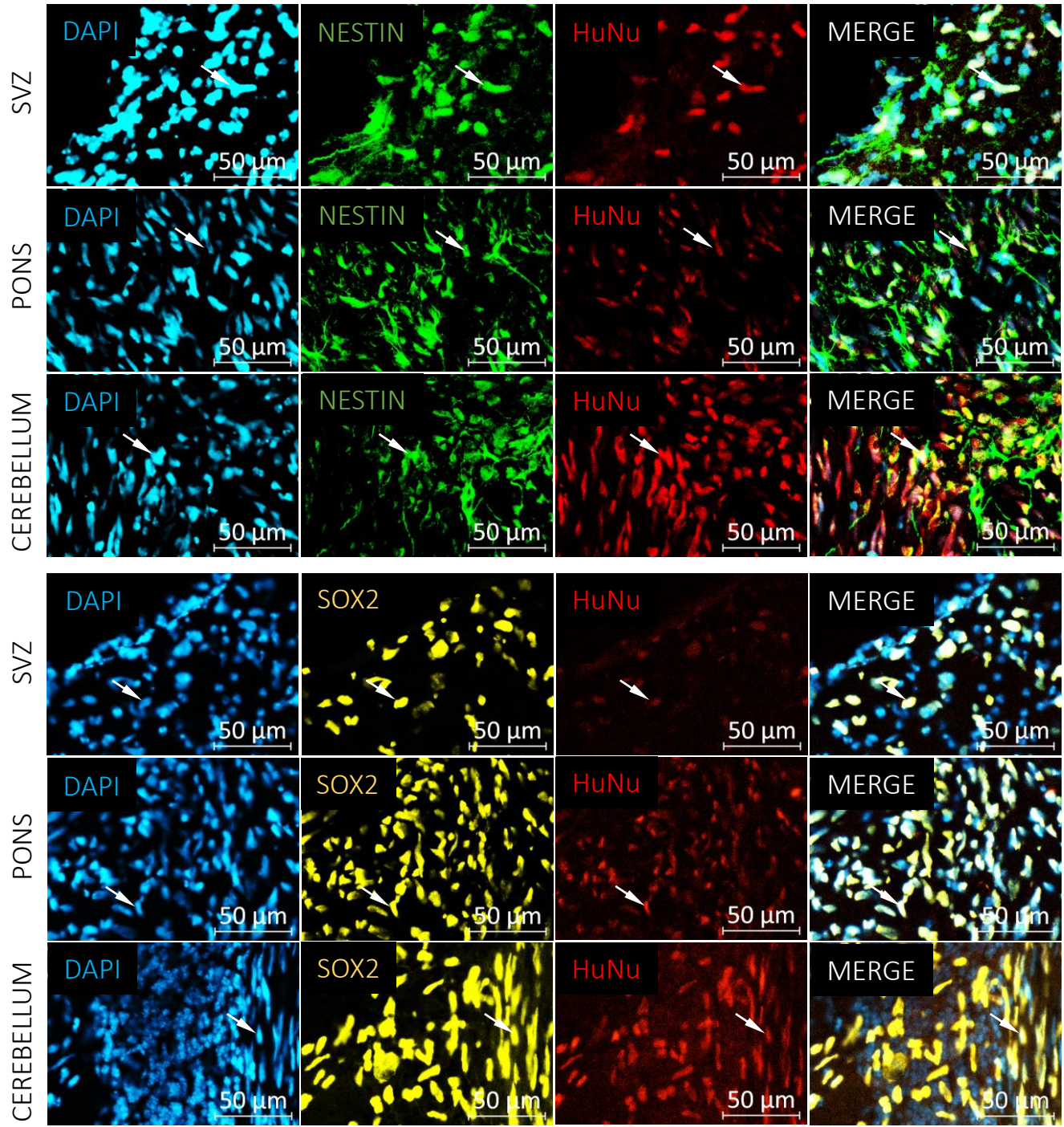

Figure S4  
(paysage)

DIPG012

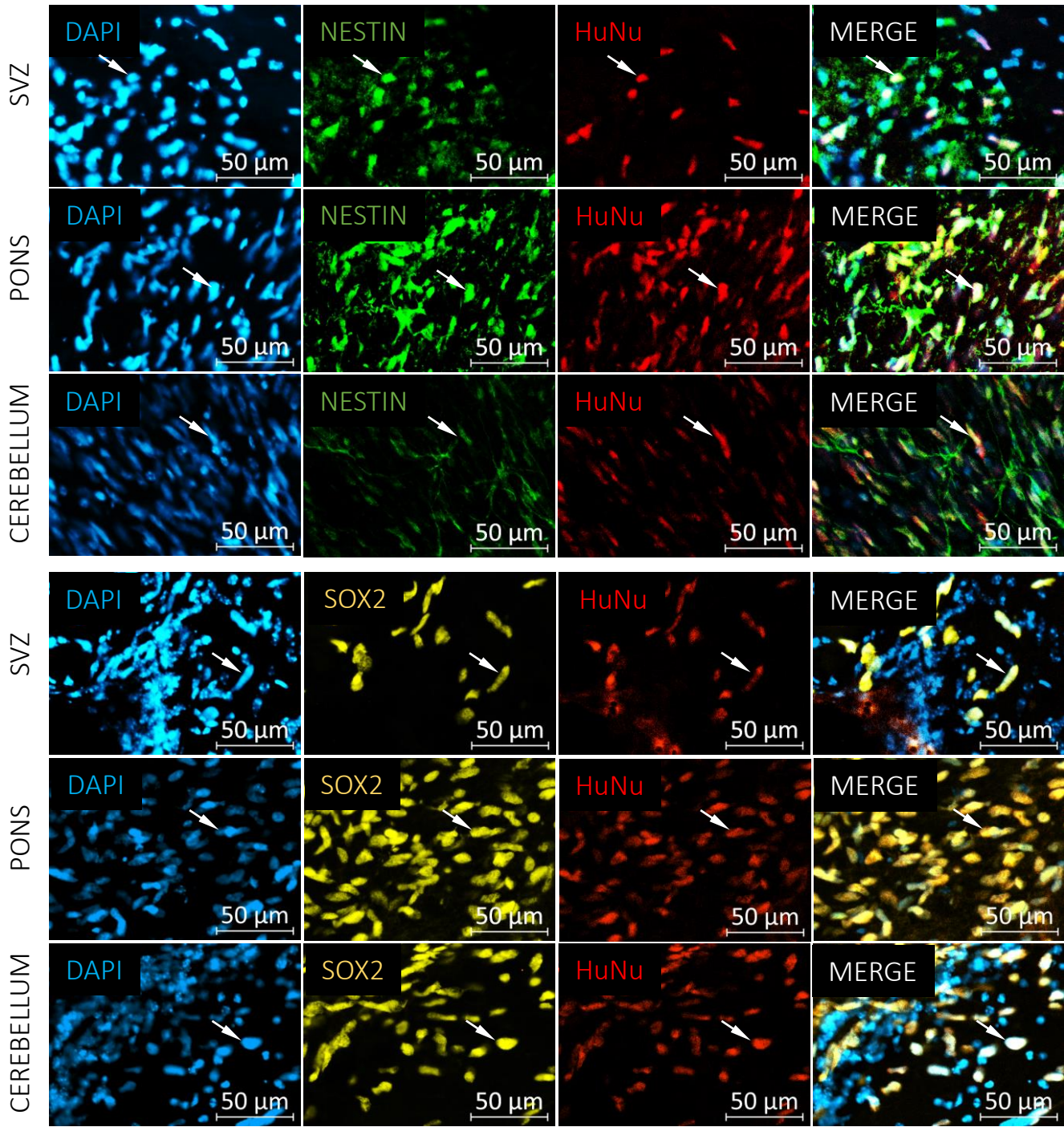

Figure S5

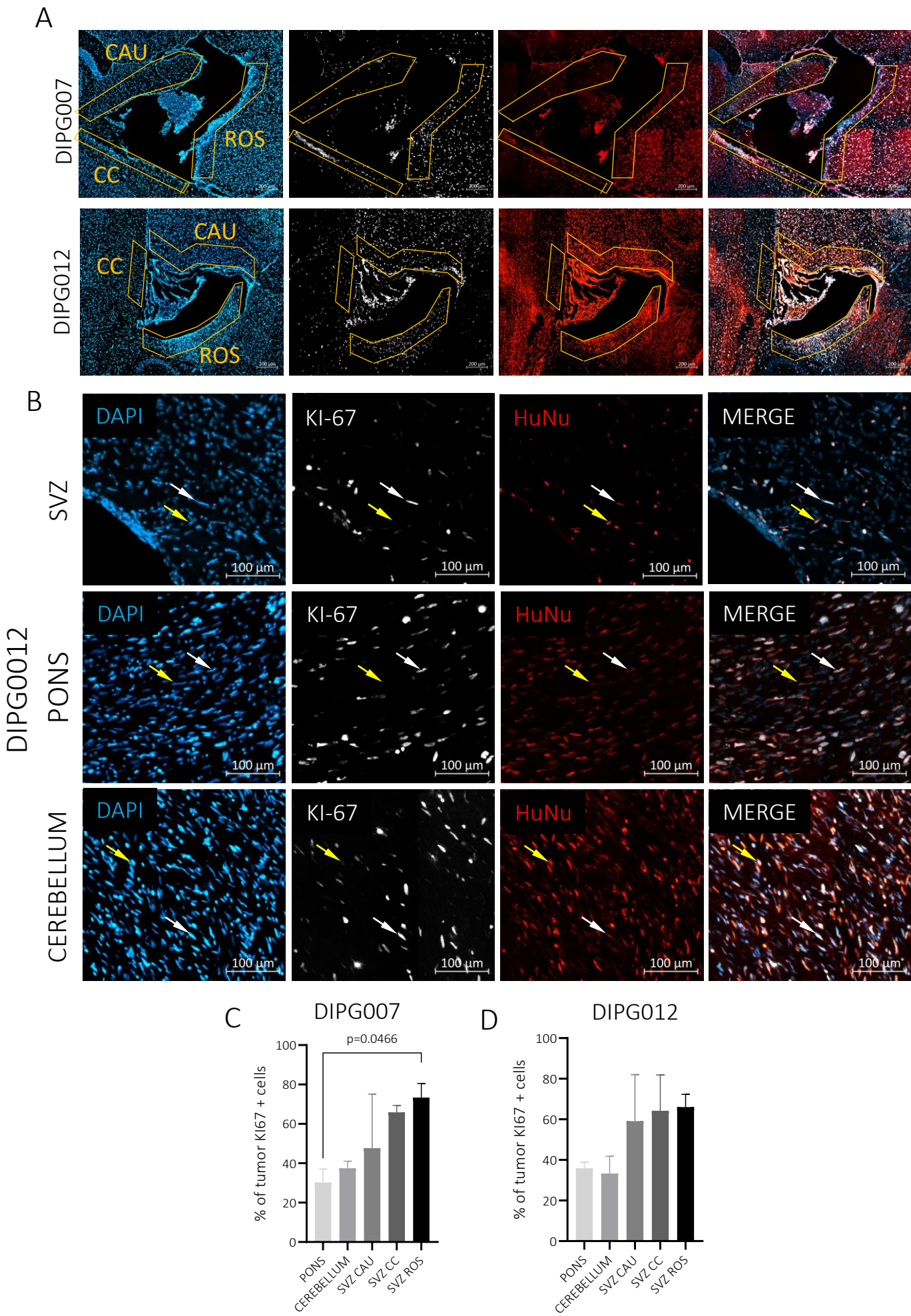

Supplement: Supplementary file 1 — Additional file 1: Fig. S1. Illustrative pictures of the limiting dilution assay. At day 7, starting with 256, 64, 16, 4 and 1 cell(s) per well. Fig. S2. Study of the quiescence/proliferation in pediatric-type glioma cell cultures by Cell Trace and EdU assays. A Mean fluorescent intensity of the CellTrace labelling decreases with time in Res259, HSJD-DIPG007, HSJD-DIPG012, in DMEM + 10% FBS as well as in NPC medium, in a similar rate (N = 3 independent experiments). B Representative phase-contrast pictures show that all three cell types are adherent in DMEM + 10% FBS, and form spheres in NPC medium. C, D Representative pictures of EdU incorporation analyzed by C epifluorescent microscopy (EdU-positive cells in red) and D via flow cytometry. White arrow indicates EdU+ cells. Fig. S3. Tumorigenicity of four selected pediatric-type glioma cell cultures upon orthotopic xenograft in mice. A Representative pictures of tumor-associated bioluminescence recorded at tumor endpoint. B Macroscopic view of Res259, KNS42, HSJD-DIPG-007 and HSJD-DIPG-012-engrafted right brain hemispheres after tissue clarification. Fig. S4. Expression of Nestin and Sox2 in DMG K27-altered cells after orthotopic xenografts. Sagittal brain sections of brains implanted with HSJD-DIPG-007 and HSJD-DIPG-012 were immunostained for anti-human nuclei (red), Nestin (green) or Sox2 (yellow) and DAPI (blue) counterstaining. Images are representative pictures of the subventricular zone (SVZ), pons and cerebellum. White arrow indicates Nestin+ or Sox2+ cells. Fig. S5. Regions of interest (ROIs) in the subventricular zone. A The subventricular zone (SVZ) was defined as the layer with 200 µm depth from the inside border of the lateral ventricle towards the brain parenchyma. SVZ was divided in three distinguishable regions: the rostral SVZ (ROS), the caudal SVZ (CAU) and the dorsal SVZ, below of the corpus callosum (CC). ROIs were established based on these parameters. B Sagittal brain sections implanted with HSJ [file 40478_2023_1586_MOESM1_ESM.pdf]
