# Supplementary material for: Comprehensive profiling of stem-like features in pediatric glioma cell cultures and their relation to the subventricular zone
Source: Acta Neuropathol Commun. 2023 Jun 16;11:96. doi: 10.1186/s40478-023-01586-x (PMC10276389; doi:10.1186/s40478-023-01586-x)
Supplement: Supplementary file 2 — Additional file 2: Table S1. List of the 95 genes sequenced by next-generation DNA sequencing. [file 40478_2023_1586_MOESM2_ESM.pdf]

List of 95 genes analyzed

AKT1	CDH1	FBXW7	H3-3A (H3F3A)	mTOR	PTCH1	SMARCA4
ALK	CDK4	FGFR1	H3-3B (H3F3B)	MYC	PTEN	SMARCB1
AMER1	CDK6	FGFR2	H3C2 (HIST1H3B)	MYCN	RAC1	SMO
APC	CDKN2A	FGFR3	H3C3 (HIST1H3C)	NF1	RAF1 (CRAF)	SPRED1
ARAF	CIC	FGFR4	HRAS	NF2	RB1	STK11
ARID1A	CTNNB1	FLT4 (VEGFR3)	IDH1	NOTCH1	RET	SUFU
ATRX	DDR2	FOXL2	IDH2	NRAS	RICTOR	TERT promotor
BAP1	DICER1	FUBP1	KIT (intron 10)	PALB2	RNF43	TP53
BCOR	EGFR	GATA3	KRAS	PDGFRA	ROS1	TSC1
BRAF	ERBB2	GLI1	LZTR1	PDGFRB	SDHA	TSC2
BRCA1	ERBB3	GLI2	MAP2K1	PIK3CA	SDHB	WT1
BRCA2	ERBB4	GNA11	MDM2	PIK3R1	SDHC	
CCND1	ERCC2	GNAQ	MDM4	POLE	SDHD	
CCNE1	ESR1	GNAS	MET (intron 13 en 14)	PRDM6	SMAD4	
