# Supplementary material for: Comprehensive profiling of stem-like features in pediatric glioma cell cultures and their relation to the subventricular zone
Source: Acta Neuropathol Commun. 2023 Jun 16;11:96. doi: 10.1186/s40478-023-01586-x (PMC10276389; doi:10.1186/s40478-023-01586-x)
Supplement: Supplementary file 3 — Additional file 3: Table S2. Antibodies used for flow cytometry experiments. [file 40478_2023_1586_MOESM3_ESM.pdf]

Table S2

Antibody	*Isotype*	*Volume per test (µl)*	*Reference*
Anti-human CD15-FITC	Mouse BALB/c IgM, κ	10	BD biosciences, 332778
Anti-human CD44-BUV737	Mouse IgG2b, κ	1	BD biosciences, 741840
Anti-human CD49f-BV421	Sprague-Dawley (outbred) IgG2a, κ	1	BD biosciences, 747725
Anti-human CD133-PE	Mouse BALB/c IgG1, κ	5	BD biosciences, 566593
Anti-human Bmi1-Alexa Fluor® 647	Mouse BALB/c IgG1, κ	5	BD biosciences, 562637
Anti-human Nestin-V450	Mouse IgG1, κ	5	BD biosciences, 561551
Anti-human Sox2-PE	Mouse IgG2a	10	BD biosciences, 560291
7-AAD Staining Solution	NA	5	BD biosciences, 559925
Fixable Viability Stain 780	NA	1	BD biosciences, 565388
