# Supplementary material for: Comprehensive profiling of stem-like features in pediatric glioma cell cultures and their relation to the subventricular zone
Source: Acta Neuropathol Commun. 2023 Jun 16;11:96. doi: 10.1186/s40478-023-01586-x (PMC10276389; doi:10.1186/s40478-023-01586-x)
Supplement: Supplementary file 4 — Additional file 4: Table S3. Antibodies used for immunofluorescent stainings on fixed cells and brain sections. [file 40478_2023_1586_MOESM4_ESM.pdf]

Table S3

Antibody	*Host*	*Concentration*	*Reference*
Anti-beta-III tubulin (Tuj1)	Mouse	1/500	Biolegend, MMS-435P
Anti-histone H3.3 K27M	Rabbit	1/400	RevMAb Biosciences, 31-1175-00
Anti-human nuclei	Mouse	1/100	Abcam, ab191181
Anti-human nuclei	Rabbit	1/200	Neobiogie, RBM5-346-P1
Anti-Ki67	Mouse	1/200	Abcam, ab185924
Anti-nestin	Chicken	1/200	Novus Bio, nb1001604
Anti-sox2	Goat	1/200	Abcam , ab239218
